# A Superimposition-Based Cephalometric Method to Quantitate Craniofacial Changes

**DOI:** 10.3390/ijerph18105260

**Published:** 2021-05-14

**Authors:** Nameer Al-Taai, Eva Levring Jäghagen, Maurits Persson, Maria Ransjö, Anna Westerlund

**Affiliations:** 1 Orthodontics, Department of Odontology, Umeå University, SE-901 85 Umeå, Sweden; maurits.persson@umu.se (M.P.); maria.ransjo@gu.se (M.R.); 2 Oral and Maxillofacial Radiology, Department of Odontology, Umeå University, SE-901 85 Umeå, Sweden; eva.levring.jaghagen@umu.se; 3 Department of Orthodontics, Sahlgrenska Academy, University of Gothenburg, SE-405 30 Gothenburg, Sweden; anna.westerlund@odontologi.gu.se

**Keywords:** cephalometry, reproducibility, skull base, superimposition

## Abstract

To assess the craniofacial changes related to growth and/or to orthodontic and orthognathic treatments, it is necessary to superimpose serial radiographs on stable structures. However, conventional superimposition provides only a graphical illustration of these changes. To increase the precision of growth and treatment evaluations, it is desirable to quantitate these craniofacial changes. The aims of this study were to (1) evaluate a superimposition-based cephalometric method to process numerical data for craniofacial growth changes and (2) identify a valid, reliable, and feasible method for superimposition. Forty pairs of cephalograms were analyzed at T1 and T2 (mean age 9.9 and 15.0 years, respectively). The superimposition-based cephalometric method involved relating the sagittal and vertical measurements on the T2 radiographs to the nasion and sella landmarks on the T1 radiographs. Validity and reliability were evaluated for three superimposition methods: the sella-nasion (SN); the tuberculum sella-wing (TW); and Björk’s structural. Superimposition-based cephalometrics can be used to quantify craniofacial changes digitally. The numerical data from the superimposition-based cephalometrics reflected a graphical illustration of superimposition and differed significantly from the data acquired through conventional cephalometrics. Superimposition using the TW method is recommended as it is valid, reliable, and feasible.

## 1. Introduction

Conventional, two-dimensional (2D) cephalometric analysis is still preferred over three-dimensional (3D) examinations for the assessment of craniofacial changes related to growth and/or to orthodontic and orthognathic treatments, since justification and minimizing of the radiation dose are priorities [1,2,3]. However, studying craniofacial changes by comparing conventional cephalometric measurements made on different occasions yields inaccurate results due to the growth-related positional changes of the used landmarks [4,5]. To assess facial changes with precision, it is necessary to superimpose serial radiographs on stable structures [6,7,8,9,10].

Cranial base superimposition is commonly performed using either (a) landmarks and planes or (b) Björk’s structural method [6]. Superimposition on the sella-nasion plane (SN) is one of the most commonly used methods [11]. However, this technique has limitations linked to the instability of the sella and nasion [6,12,13,14]. The use of the tuberculum sella-wing (TW) plane for superimposition has been reported to be more advantageous [15], since the T and W landmarks reach stability at an early age [14,16]. Meanwhile, Björk’s structural method takes advantage of the fact that the cranial base acquires 90% of its final size by 4–5 years of age [13] and represents a stable reference for superimposition [6,7,10,12,14,17,18]. This latter method has been considered as the “gold standard” and has been validated in a histologic study conducted by Melsen [14]. The difficulties associated with identifying the structures using Björk’s method can be mitigated through the use of subtraction radiography, which allows for optimal orientation of the radiographs [19,20]. With the advent of the digital era, the subtraction technique has become more accessible, although few studies have assessed its performance [19,20,21].

Several research groups have compared the levels of reliability and validity of cranial base superimposition methods, with variable outcomes [15,17,18,19,21,22,23,24]. Some of these groups have proposed the use of the structural method based on its validity [6,7,12,14,16,18,19]. Other groups have claimed that the use of the landmark method (SN) does not give significantly different results from those obtained with the structural method, and they have therefore recommended the SN method, owing to its high levels of reliability and feasibility [22,23,24]. Thus, it remains unclear as to which method is optimal. Furthermore, conventional superimposition provides only a graphical illustration of the craniofacial changes occurring over time. To increase the precision of growth and treatment evaluations, it is desirable to quantitate these craniofacial changes.

We hypothesized that the superimposition-based cephalometric method using a valid and reliable superimposition method would provide numerical data reflecting craniofacial changes related to growth and treatments outcomes.

The overall aims of this study were to (1) evaluate a digital superimposition-based cephalometric method to acquire numerical data that reflect the craniofacial changes related to growth and treatment outcomes and (2) identify a valid, reliable, and feasible superimposition method that can be used as the basis for superimposition-based cephalometrics.

## 2. Materials and Methods

### 2.1. Subjects

This methodological study was conducted according to the guidelines of the Declaration of Helsinki and was approved by the Ethics Committee at Umeå University (Dnr. 2012-410-31M), which waived the requirement to obtain informed consent from all subjects because the material for this study was collected more than 50 years ago.

Forty pairs of cephalometric radiographs acquired from growing and untreated boys (N = 20) and girls (N = 20), with a mean time interval of 5 years from baseline (T1) to follow-up (T2), were selected. The mean ages of the boys and girls were 10.3 ± 0.83 and 9.6 ± 0.69 years, respectively, at T1, and 15.3 ± 0.84 and 14.9 ± 0.69 years, respectively, at T2. This age range was chosen to include the growth spurt and to take advantage of the major growth changes. There were 20 individuals with Class I, 13 with Class II, and 7 with Class III skeletal malocclusions. The patient material was derived from standardized cephalometric radiographs [25]. The radiographs were high-quality analog cephalograms and all had been acquired using the same cephalostat.

The radiographs were scanned using the Epson Perfection V750 Pro scanner with a resolution of 250 dpi (0.092120 mm/pixel). They were imported as JPEG files into the digital analysis program FACAD^®^ (cephalometric software ver. 3.9.2.1133; Ilexis AB, Linköping, Sweden), where they were standardized and analyzed.

### 2.2. Landmarks and Reference Lines

**Figure 1:**
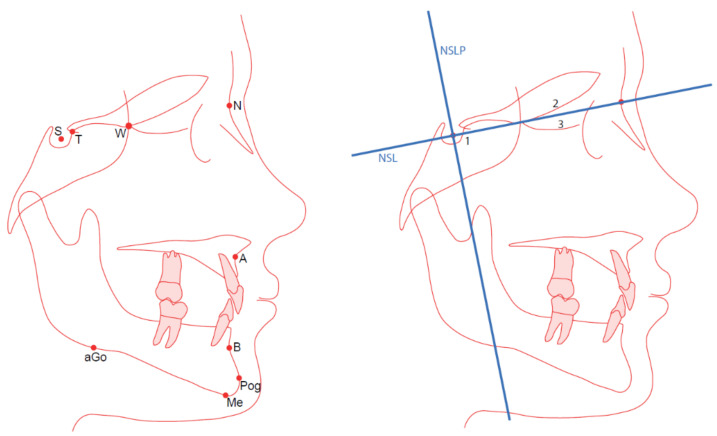
(**Left panel**) The used landmarks are S, sella; T, tuberculum sella (Walker’s point); W, wing point (sphenoethmoidal); N, nasion; A, A-point; B, B-point; Pog, pogonion; Me, menton; aGo, anterior gonion. (**Right panel**) The reference lines (NSL and NSLP) and superimposition using the Björk’s BD, BT, and BS methods, performed on the 1, anterior sella; 2, cribriform plate; and 3, ethmoidal crest.

Nine landmarks (S, T, W, N, A, B, Pog, Me, and aGo) were used in this study (Figure 1).

Two reference lines, the nasion-sella line (NSL) and a perpendicular line through the sella (NSLP), were used as reference lines (Figure 1). Landmarks S and N were used as reference landmarks.

Fifteen variables (linear and angular measurements), measured in relation to the reference lines or landmarks, were used in this study:

- Five vertical distances, from the landmarks (N, A, B, Pog, Me) to the horizontal reference line NSL;
- Five horizontal distances, from the landmarks (N, A, B, Pog, Me) to the vertical reference line NSLP;
- Three angles (SNA, SNB, ANB) representing the sagittal relation; and
- One angle (ML/NSL) and one linear height (N-Me) representing the vertical relation.

### 2.3. Different Methods for Growth Evaluation

#### 2.3.1. Conventional Cephalometrics

**Figure 2:**
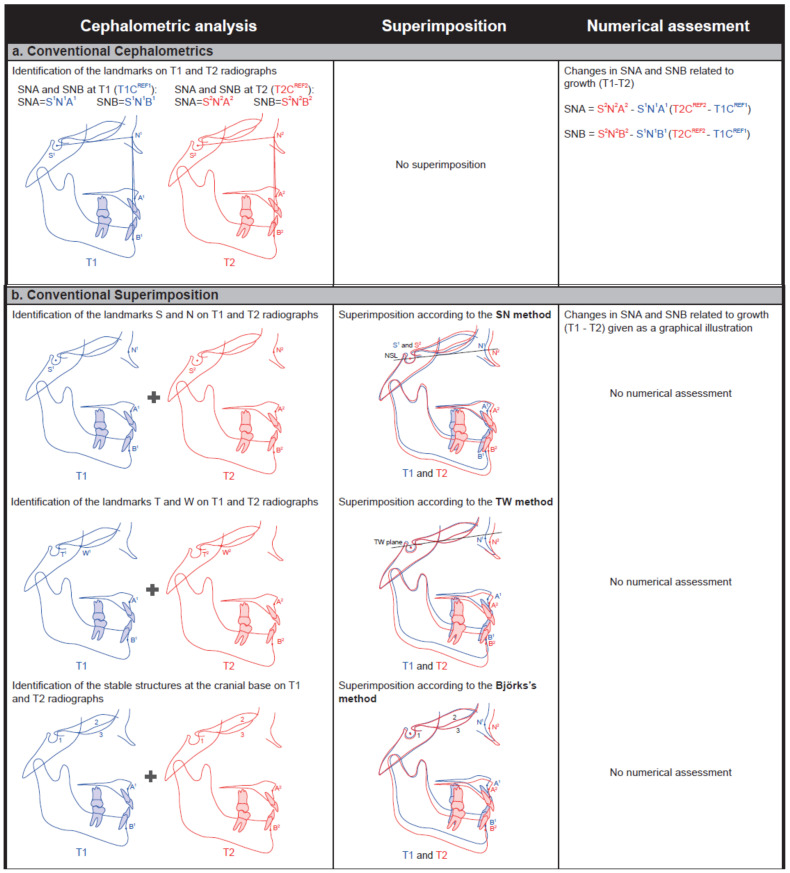

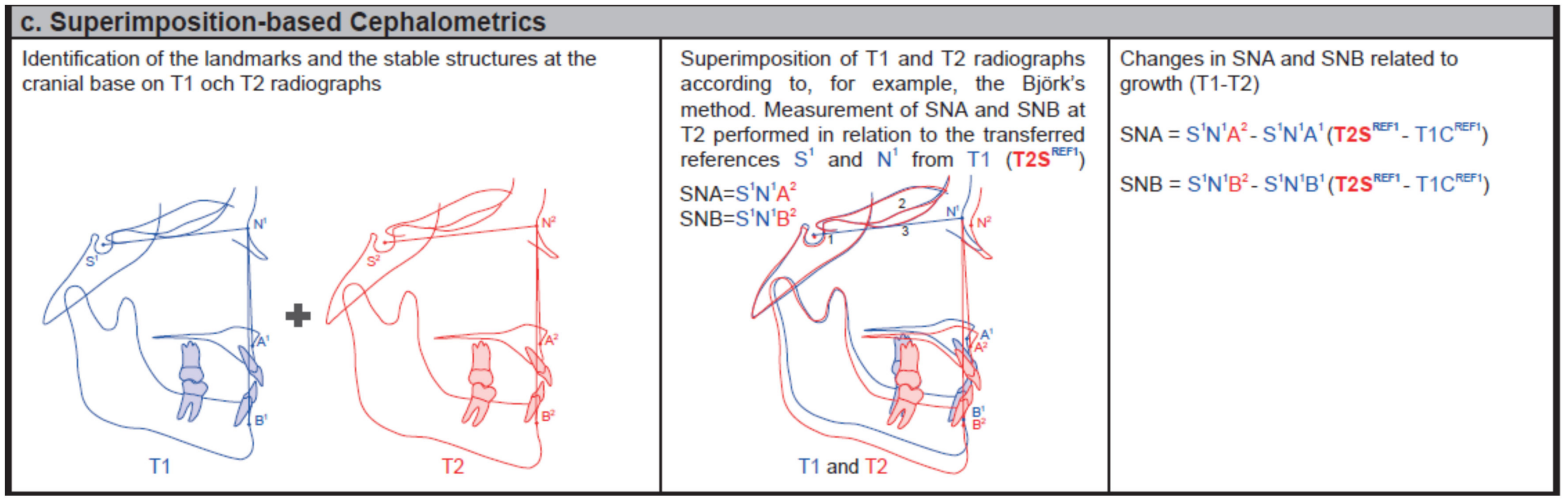
Evaluations of craniofacial changes related to growth and treatment (e.g., SNA and SNB) are performed using three methods: (**a**), conventional cephalometrics; (**b**), conventional superimposition; and (**c**), superimposition-based cephalometrics.

The measurements of the 15 variables on the T1 radiographs, performed in relation to the T1 references NSL, NSLP, S, or N, were designated as T1C^REF1^. The measurements of the 15 variables on the T2 radiographs, performed in a similar manner but in relation to the T2 references, were designated as T2C^REF2^. Changes related to the growth from T1 to T2 were calculated as the difference between the 15 variables at T1 and T2 (T2C^REF2^ − T1C^REF1^) (Figure 2a).

#### 2.3.2. Conventional Superimposition

Radiographs from T1 and T2 were superimposed on landmarks (SN method or TW method) or structures (Björk’s structural method). Changes related to growth from T1 to T2 are given solely as a graphical illustration (Figure 2b).

#### 2.3.3. Superimposition-Based Cephalometrics (New Method)

**Figure A1:**
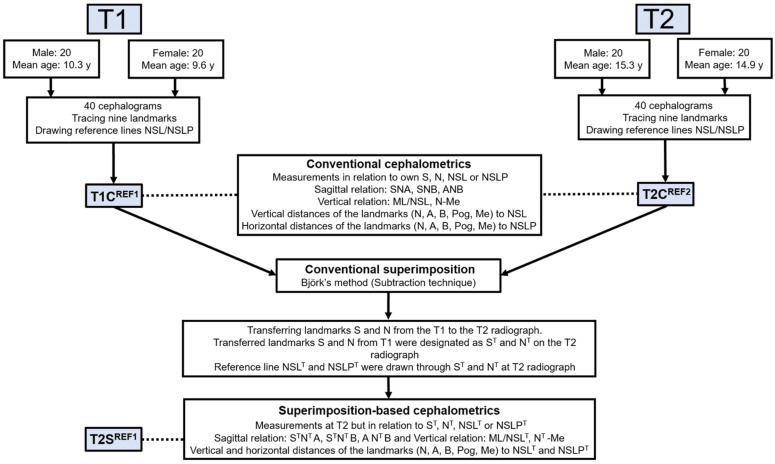
Workflow for the superimposition-based cephalometrics (new method).

To quantitate the actual craniofacial changes related to growth, it is necessary to superimpose on stable structures. Thus, Björk’s structural method (subtraction technique) was selected for the superimposition of radiographs T1 and T2 according to the protocol in Part II (see below). Landmarks S and N and reference lines NSL and NSLP were transferred digitally from radiograph T1 to radiograph T2 (designated as S^T^, N^T^, NSL^T^, and NSLP^T^, respectively). Thereby, each T2 radiograph, besides having its own references S, N, NSL, and NSLP, also had S^T^, N^T^, NSL^T^, and NSLP^T^ references with the same positions as those in T1. The digital software facilitated measurements of the 15 variables on each T2 radiograph in relation to these transferred landmarks and reference lines (designated as T2S^REF1^). Relating the measurements of the 15 variables at T2 to the reference lines or landmarks at T1 required superimposition. Therefore, we refer to this as superimposition-based cephalometrics (Appendix A, Figure A1). Changes related to the growth from T1 to T2 were calculated as the difference between the 15 variables (T2S^REF1^ − T1C^REF1^) (Figure 2c).

### 2.4. Part I. Evaluation of the Superimposition-Based Cephalometric Method

To determine the importance of superimposition-based cephalometrics, we investigated whether there were differences between superimposition-based cephalometrics and conventional cephalometrics. Thus, measurements, of five vertical and sagittal variables (ML/NSL, N-Me and SNA, SNB, ANB) were performed using the two methods, the conventional cephalometric method (T2C^REF2^) and the superimposition-based cephalometric method (T2S^REF1^), on the same occasion (T2). In addition, the growth-related positional changes of the nasion were evaluated (T1–T2), using the T2S^REF1^ measurement, to investigate whether these changes affect the evaluation of the five vertical and sagittal variables.

### 2.5. Part II. Evaluation of the Different Conventional Superimposition Methods

To identify a valid, reliable, feasible and digital superimposition method that would serve as the basis for the superimposition-based cephalometrics, the following three cranial base superimposition methods were evaluated:

- The Steiner method (SN) [11];
- The tuberculum sella-wing method (TW) [15];
- Björk’s structural method, performed using three techniques:
  1. direct (BD) [6,21];
  2. tracing template (BT) [17,21]; and
  3. subtraction (BS) [19,20].

Björk’s structural method with these three techniques was performed on the following stable structures: (i) the anterior part of the sella turcica, to orient the radiographs in a horizontal direction; (ii) the cribriform plate; and (iii) the ethmoidal crest, to orient the radiographs in a vertical direction (Figure 1) [13,14,22].

To evaluate these superimposition methods, superimposition-based cephalometrics (T2S^REF1^) were used. Fifteen variables were produced for each T2 radiograph and for each superimposition method, i.e., 15 variables were measured in relation to the references NSLT, NSLPT, ST, or NT, which were transferred from the T1 radiograph after each superimposition method.

#### 2.5.1. Validity

The growth-related facial changes (T1–T2), for the 40 radiographs, were evaluated by the SN, TW, and Björk’s methods. The changes in the measurements (T2S^REF1^ − T1C^REF1^) of all 15 variables were compared across the methods in order to evaluate the validities of the SN and TW methods, as compared with Björk’s method (three techniques).

#### 2.5.2. Reliability

To assess the intra-observer reliability of each method, the superimpositions with all the methods were repeated by a single orthodontist (N.A.-T.) on two separate occasions 3 weeks apart. The differences in the T2S^REF1^ measurements of the 10 variables between the two occasions were calculated. Superimpositions with all the methods were performed by another orthodontist (A.W.) at a single sitting on 10 randomly selected radiographs to determine the inter-observer reliability of each method. The variables used to assess the reliability were the vertical and horizontal distances from the landmarks (N, A, B, Pog, Me) to the horizontal (NSL) and vertical (NSLP) reference lines, respectively.

#### 2.5.3. Workflow—Evaluations of the Different Superimposition Methods (Reliability, Validity)

**Figure A2:**
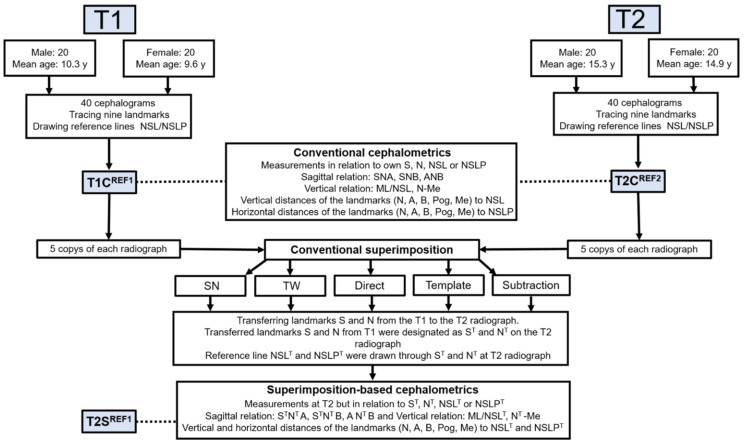
Workflow for the different superimposition methods.

To ensure that landmark identifications were consistently applied across all the methods, five digital copies of each radiograph were created after landmark identification, i.e., one copy for each method and time-point. Thus, the measurements of the 15 variables, T1C^REF1^ and T2C^REF2^, were standardized before superimposition for all five methods at both T1 and T2 (Appendix A, Figure A2).

The superimposition protocol for the 40 radiographs was as follows:

- SN: Radiographs T1 and T2 were superimposed on the NSL with registration at the sella.
- TW: The TW line was drawn through landmarks T and W on the T1 and T2 radiographs. Radiographs T1 and T2 were superimposed on the TW line with registration at landmark T.
- BD: Radiographs T1 and T2 were superimposed directly, using as good a fit as possible on the anterior part of the sella turcica, the cribriform plate, and the ethmoidal crest.
- BT: The anterior part of the sella turcica, the cribriform plate, and the ethmoidal crest on the T1 radiograph were drawn on a template. Thereafter, the template was superimposed on the T2 radiograph, ensuring the best possible fit for these three structures.
- BS: A positive copy of the T1 radiograph was created. This copy was thereafter superimposed on the T2 radiograph, to subtract details around the anterior contour of the sella turcica, the cribriform plate, and the ethmoidal crest.

Superimposition using all the different methods was performed on one subject before proceeding to the next subject. One orthodontist (N.A.-T.), who has more than 8 years of experience with superimposition, performed the tracing and superimposition digitally.

### 2.6. Intra- and Inter-Observer Method Errors

To study the intra-observer reliability of the cephalometric measurements used, tracings of 20 randomly selected radiographs (10 at T1 and 10 at T2; 12 boys and 8 girls) were performed by a single orthodontist (N.A.-T.) on two occasions, with a 3-week interval. Tracings of these 20 radiographs were performed again by another orthodontist (A.W.) at a single sitting, to study the inter-observer reliability levels of the cephalometric measurements. The intra- and inter-observer reliability levels were assessed by estimating the intra-class correlation coefficients (ICCs) with 95% confidence intervals. The intra- and inter-observer reliabilities of the cephalometric measurements were good, with ICCs in the ranges of 0.96–0.99 and 0.91–0.99, respectively.

### 2.7. Statistical Analysis

A paired *t*-test was used to determine if there were significant differences in the sagittal and vertical measurements between the T2C^REF2^ and T2S^REF1^.

To evaluate the validity of the methods, the systematic differences between the superimposition methods were assessed using repeated-measures ANOVA, which was used to compare the mean differences for the 15 variables (T2S^REF1^ − T1C^REF1^) between the various methods applied.

A post-hoc test was performed using Tukey’s honest significant difference test to adjust for pairwise comparisons of the results obtained with the different superimposition methods.

The statistical significance level was set at *p* < 0.05. Statistical analyses were performed using the SPSS for Windows software.

The intra- and inter-observer reliabilities of the superimposition methods were assessed by estimating the intra-class correlation coefficients (ICC) with 95% confidence intervals, which were established using one-way and two-way random effects models, respectively.

## 3. Results

### 3.1. Part I. Evaluation of the Superimposition-Based Cephalometric Method

Comparison of conventional cephalometrics (T2C^REF2^) and superimposition-based cephalometrics (T2S^REF1^) showed significant differences for the sagittal (SNA, SNB, ANB) and vertical (N-Me) relations (four out of five compared variables) (Table 1). Investigation of the positional changes of the nasion showed that, horizontally, the nasion was significantly displaced forward (by about 4 mm) in all the subjects, when comparing T1 and T2 (Table 2). Significant differences were found among the methods, as the SN method differed from the other methods (Table 3). Vertically, the nasion was displaced downwards in 34 subjects and upwards in 16 subjects from T1 to T2, with no significant differences noted between T1 and T2 or among the superimposition methods.

### 3.2. Part II. Evaluations of the Different Superimposition Methods

Regarding validity, significant differences were found between the SN method and the other methods with respect to the horizontal positional changes of landmarks (A, B, N), sagittal angular measurements (SNA, SNB, ANB), and a vertical measurement (N-Me) (Table 2 and Table 3). In contrast, there were no significant differences for any of the examined variables between the TW method and Björk’s three techniques (Table 3).

**Figure 3:**
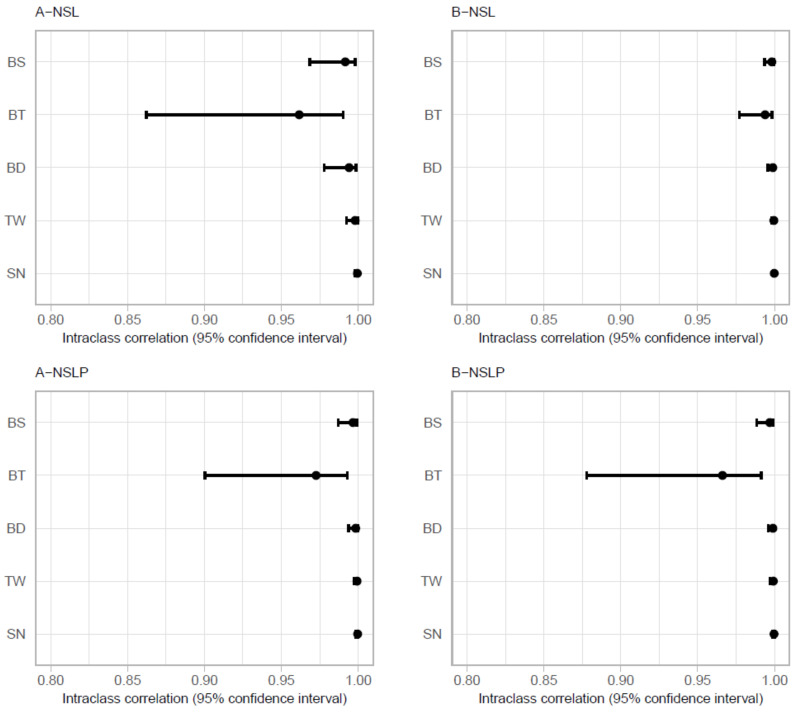
ICC values for the BD, BT, BS, TW, and SN methods presented for four variables: vertical and horizontal distances of the landmarks A and B and to the horizontal (NSL) and vertical (NSLP) reference lines, respectively.

Investigation of the intra-observer reliability levels for all the studied superimposition methods assessed with the ICCs revealed a high level of agreement for the examined variables (ICC > 0.95), with SN and TW having markedly high ICC values (Table 4, Figure 3). Regarding the levels of reliability of Björk’s three techniques, the BT method had a lower estimated ICC than the BS and BD methods, although the respective 95% confidence intervals overlapped (Table 4, Figure 3). The inter-observer reliability levels for all the studied methods showed a high level of agreement for all the examined variables (ICC > 0.95), with the exception of the vertical distance of the nasion to the horizontal reference line (N-NSL), which showed a good level of agreement for the SN and TW methods and poor agreement between observers for Björk’s techniques (BD, BT, and BS) (Table 5). The relatively low values of ICC for N-NSL are explained by very low variance between radiographs for this variable.

## 4. Discussion

Numerical data from conventional two-time-point cephalometry lack accuracy due to the growth-related displacement of landmarks, especially in relation to the nasion, while conventional superimposition on stable structures renders only a graphical illustration of the craniofacial changes. This study presents a superimposition-based cephalometric method that generates numerical data for the actual craniofacial changes occurring over time. The underlying concept is to superimpose on stable structures and, thereafter, use the same reference landmarks S and N when measuring the sagittal and vertical relations in the baseline (T1) and follow-up (T2, T3, etc.) radiographs. That the method is convenient is demonstrated by the finding that the numerical data derived from superimposition-based cephalometrics, unlike conventional cephalometrics, reflect the graphical illustration of the superimposition.

Based on the comparisons of the different superimposition methods, we found that the TW method is as valid as Björk’s structural method and is as reliable and feasible as the SN method. Therefore, we consider the TW method to be the most suitable strategy for cranial base superimposition. Furthermore, it can be used rather than Björk’s method for superimposition-based cephalometrics.

Positional changes of landmarks (especially of the nasion), which are related to growth, have a crucial impact on the assessment of facial changes when using conventional cephalometrics. In the present study, we demonstrated significant forward displacement of the nasion from T1 to T2. This results in significant differences between conventional cephalometrics and superimposition-based cephalometrics, even though both measurements are performed at T2. The use of conventional cephalometrics yielded an underestimation of the sagittal growth in all the subjects due to the forward displacement of the nasion. No significant movement of the nasion in the vertical direction could be demonstrated, explained as an upward shift for some of the patients and a downward shift for most of patients. Consequently, the vertical growth was underestimated or overestimated for patients with a downward or upward shift of the nasion, respectively. To avoid underestimation of sagittal growth and underestimation and overestimation of vertical growth, landmarks N and S must be kept constant. This can be achieved using superimposition-based cephalometrics, by employing the S and N from T1 as stable references for the subsequent T2 measurements. Thus, one can argue that changes in, for example, the SNA angle at T2 depend exclusively on the horizontal and/or vertical displacement of landmark A. Furthermore, by assessing the positional changes of landmark A at T2 in relation to the reference lines (NSL^T^ and NSLP^T^) transferred from T1, it is possible to quantitate and interpret the precise sagittal changes of the maxillary apical base. The same concept applies to the other angular and linear measurements related to the landmarks N and/or S, e.g., SNB, ANB, SNPog, SNBa, SNAr, N-Ba, N-Me, ML/NSL, NL/NSL and ILs/NSL, which can be used to quantitate and interpret the craniofacial changes related to growth and/or orthodontic and orthognathic treatments.

The reliability and validity levels of a superimposition method are dependent upon the accurate identification of landmarks and structures [17]. These, in turn, rely on the quality of the radiographs and digitization, the skill and experience of the operator, and the specific superimposition method used [21]. While some studies have found no significant differences between manual and digital tracing and superimposition [26,27], other studies have shown that digital tracing improves the identification of landmarks, thereby improving the accuracy of the superimposition [28,29].

Most of the studies conducted to date have shown that Björk’s structural method of superimposition, using stable structures in the cranial base, has the highest level of validity [6,7,12,14,15,16,17,18]. Since the present study shows no significant differences in the 15 variables between the TW method and all Björk’s techniques, superimposition using the TW plane method can be considered to be as valid as using Björk’s structural method. This is in agreement with the results from a study conducted by Arat and coworkers [15]. In that study, however, the superimposition was performed manually, and other statistical methods were used. Furthermore, they did not study the vertical and sagittal angular relations [15].

**Figure 4:**
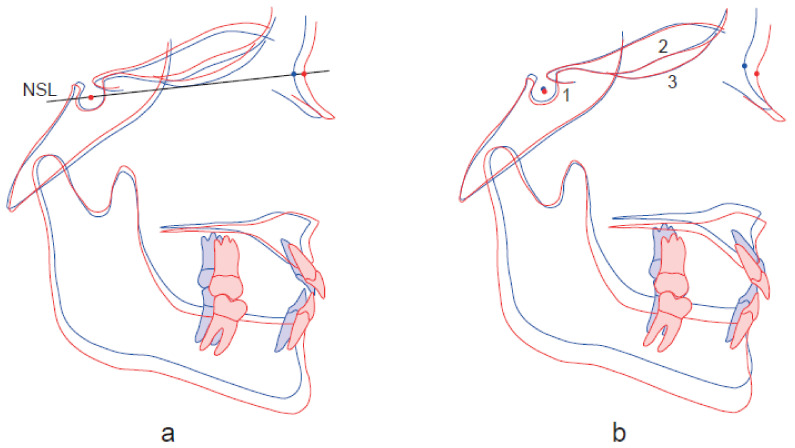
Illustration of how superimposition using the SN method (**a**) overestimates the sagittal relation and underestimates the vertical relation, due to displacement of the nasion (T1 T2) forwards and downwards, as compared to the superimposition-based cephalometrics using Björk’s structural method (**b**).

Despite the observed high-level reliability, the results of our study confirm that superimposition using the SN method has low validity in terms of interpretation of the sagittal and vertical relations (Figure 4), and this is attributed to the instability of the S and N landmarks [6,9,12,14,16,18]. We observe significant differences between the SN method and the other methods in the horizontal positional changes of landmarks A, B, and N and in the sagittal (SNA, SNB, ANB) and vertical (N-Me) relations.

Our results show that the SN method has high reliability, in agreement with what was reported in previous studies [9,21]. However, we show that all the methods have high levels of reliability (ICC values > 0.95). In particular, SN and TW show high intra- and inter-observer reliability. The double images observed for the T and W landmarks and the tracing of an equidistant point do not seem to affect the reliability of the TW method.

We have observed that it is easy to identify landmarks T and W and to perform the superimposition digitally using the TW method. In contrast, performing Björk’s superimposition with one of the three studied techniques is time-consuming, both in terms of identifying the stable structures and orienting the radiographs, particularly with respect to the cribriform plate and the ethmoidal crest. Of Björk’s three techniques, we recommend the subtraction technique, as it has a higher ICC compared with that of the template technique and it is easier to perform.

The discrepancy between the previous studies that showed no significant differences between the SN method and Björk’s structural method [22,23,24] and our finding that there is a difference between these two methods can be explained by differences in material selection, statistical analysis, follow-up times, and evaluation methods. For example, Lenza and coworkers did not present the sagittal and vertical relations and their follow-up period did not cover the growth spurt [22]. Furthermore, they used a one-way ANOVA test, ignoring the repeated measurements of the same subjects, thereby risking a loss of power. In the earlier studies [23,24], the tracings and superimposition were carried out manually, the sample sizes were smaller, and the follow-up period times were shorter (3 years and 7.5 months, respectively). Moreover, their subjects were followed primarily to evaluate orthodontic treatment rather than to study growth. In our study, the timing of the follow-up (T1–T2) was chosen to capture the growth spurt during puberty for both boys and girls [30].

Cone beam computed tomography (CBCT) with 3D imaging has introduced advanced opportunities to perform surface and voxel-based superimposition, making analyses of, e.g., volumetric craniofacial and pulp chamber changes possible [31,32,33,34]. CBCT for superimposition has shown good precision and reliability [33,34].

A limitation to this study is the usage of 2D images for landmark identification and measurements of 3D craniofacial changes, which can result in errors [10]. However, identifying stable landmarks remains a challenge for both 2D and 3D methods and a considerably higher radiation dose, up to 26-fold higher, has been reported for large field of view CBCT examinations, as compared to a lateral cephalogram [2,3]. Such a level of radiation exposure can only be justified in specific cases, not in routine orthodontic practice and especially not if multiple repeated examinations are necessary. Therefore, 2D cephalometric analysis remains the method of choice when assessing craniofacial changes related to growth and/or orthodontic and orthognathic treatments in daily practice.

## 5. Conclusions

Superimposition-based cephalometrics can be used to quantitate precisely craniofacial changes that occur over time. Using stable landmarks, superimposition-based cephalometrics reflects accurately the graphical illustration of the superimposition.

The TW method is a valid, reliable, and feasible superimposition method that can be used as the basis for superimposition-based cephalometrics.

## Figures and Tables

**Table 1:** Mean (standard deviation) differences between conventional cephalometrics (T2C^REF2^) and superimposition-based cephalometrics (T2S^REF1^) (N = 40).

Variables	Mean (SD)	*p*-Value ^1^
SNA (°)	3.64 (2.62)	<0.001
SNB (°)	2.28 (1.67)	<0.001
ANB (°)	1.36 (1.01)	<0.001
ML/NSL (°)	0.04 (0.76)	0.71
N-Me (mm)	−0.45 (1.11)	0.014

^1^ Calculated using paired *t*-tests to compare the two methods.

**Table 2:** Mean changes (standard deviation) for the 15 variables of the whole sample (T2S^REF1^ − T1C^REF1^) compared for the superimposition methods: SN, TW, BD, BT, and BS.

Variables	SN	TW	BD	BT	BS	*p*-Value ^1^
A-NSL (mm)	6.4 (2.3)	6.6 (2.3)	6.4 (2.1)	6.6 (2.2)	6.6 (2.3)	0.61
B-NSL (mm)	8.5 (3.2)	8.8 (3.2)	8.6 (3.2)	8.7 (3.2)	8.8 (3.3)	0.489
N-NSL (mm)	0.0 (0.085)	0.18 (1.5)	0.063 (0.94)	0.20 (1.6)	0.27 (0.95)	0.707
Pog-NSL (mm)	11 (3.6)	11 (3.6)	11 (3.6)	11 (3.6)	11 (3.6)	0.458
Me-NSL (mm)	11 (3.8)	11 (3.8)	11 (3.7)	11 (3.8)	11 (3.8)	0.337
A-NSLP (mm)	4.7 (2.1)	4.3 (2.2)	4.1 (2.2)	3.9 (2.1)	3.9 (2.2)	<0.001 ***
B-NSLP (mm)	4.4 (3.2)	4.1 (3.0)	3.8 (3.1)	3.5 (3.0)	3.6 (3.2)	0.0351 *
N-NSLP (mm)	4.4 (2.6)	3.9 (2.6)	3.7 (2.6)	3.8 (2.5)	3.7 (2.5)	<0.001 ***
Pog-NSLP (mm)	5.3 (3.9)	4.9 (3.5)	4.6 (3.7)	4.3 (3.5)	4.5 (3.8)	0.0693
Me-NSLP (mm)	5.3 (4.0)	4.9 (3.7)	4.6 (3.8)	4.3 (3.6)	4.4 (3.8)	0.0931
SNA (°)	1.3 (2.9)	5.3 (2.3)	5.0 (2.2)	4.9 (2.1)	4.9 (2.3)	<0.001 ***
SNB (°)	1.2 (2.4)	3.7 (1.9)	3.5 (2.0)	3.4 (1.9)	3.4 (2.0)	<0.001 ***
ANB (°)	0.12 (1.3)	1.6 (1.3)	1.5 (1.3)	1.5 (1.3)	1.5 (1.3)	<0.001 ***
ML/NSL (°)	−2.7 (3.0)	−2.8 (2.9)	−2.7 (3.0)	−2.5 (2.6)	−2.7 (2.9)	0.768
N-Me (mm)	11 (4.0)	10 (3.6)	10 (3.5)	10 (3.5)	10 (3.6)	0.0176 *

^1^ Calculated using repeated-measures ANOVA to evaluate the differences between the methods. Statistically significant differences at * *p* < 0.05 and *** *p* < 0.001.

**Table 3:** The *p*-values from pairwise post-hoc tests, adjusted for multiple comparisons within each variable using Tukey’s honest significant difference method (N = 40) for the sagittal and vertical relations across five superimposition methods (T2S^REF1^ − T1C^REF1^), showing that the SN method differs from the other methods.

Variables	TW-SN	BD-SN	BT-SN	BS-SN	BD-TW	BT-TW	BS-TW	BT-BD	BS-BD	BS-BT
A-NSLP	0.316	0.017	0.001	0.002	0.729	0.291	0.335	0.951	0.969	1.000
B-NSLP	0.805	0.266	0.045	0.081	0.894	0.437	0.586	0.933	0.981	0.999
N-NSLP	<0.001	<0.001	<0.001	<0.001	0.079	0.260	0.066	0.981	0.907	0.609
SNA	<0.001	<0.001	<0.001	<0.001	0.950	0.828	0.842	0.997	0.998	1.000
SNB	<0.001	<0.001	<0.001	<0.001	0.954	0.698	0.853	0.978	0.998	0.999
ANB	<0.001	<0.001	<0.001	<0.001	0.967	0.999	0.911	0.995	1.000	0.975
N-Me	0.051	0.016	0.080	0.185	0.995	1.000	0.981	0.978	0.875	0.996

**Table 4:** Intra-observer reliability levels of all the studied superimposition methods (SN, TW, BD, BT, and BS), assessed as the ICCs for 10 variables.

	A-NSL	B-NSL	N-NSL	Pog-NSL	Me-NSL	A-NSLP	B-NSLP	N-NSLP	Pog-NSLP	Me-NSLP
**SN**	0.999	1.000	0.000 *	1.000	1.000	1.000	1.000	1.000	1.000	1.000
**TW**	0.998	1.000	0.990	1.000	1.000	0.999	0.999	1.000	0.999	0.999
**BD**	0.994	0.999	0.934	0.999	0.999	0.998	0.999	0.999	0.999	0.998
**BT**	0.962	0.994	0.846	0.994	0.996	0.973	0.966	0.998	0.959	0.953
**BS**	0.992	0.998	0.879	0.998	0.999	0.997	0.997	0.997	0.996	0.995

* Calculation resulted in a negative value, which was set to zero.

**Table 5:** Inter-observer reliability levels of all the studied superimposition methods (SN, TW, BD, BT, and BS), assessed as the ICCs for 10 variables.

	A-NSL	B-NSL	N-NSL	Pog-NSL	Me-NSL	A-NSLP	B-NSLP	N-NSLP	Pog-NSLP	Me-NSLP
**SN**	0.912	0.991	0.862	0.991	0.997	0.987	0.985	0.988	0.985	0.980
**TW**	0.925	0.987	0.760	0.981	0.991	0.963	0.955	0.977	0.966	0.962
**BD**	0.970	0.994	0.492	0.993	0.994	0.994	0.997	0.991	0.997	0.997
**BT**	0.838	0.972	0.000 *	0.970	0.977	0.962	0.960	0.990	0.952	0.943
**BS**	0.975	0.995	0.483	0.995	0.995	0.994	0.997	0.990	0.997	0.997

* Calculation resulted in a negative value, which was set to zero.

## Data Availability

The data presented in this study are available on request from the corresponding author.
